# Supposed Poisoning by Hydrate of Chloral

**Published:** 1871-02

**Authors:** 


### SUPPOSED POISONING BY HYDRATE OF CHLORAL.

Some details of a case of supposed poisoning by this popular hypnotic have been sent us by a correspondent in Illinois. The statement is as follows :

"On the morning of the 25th, Mrs. Fletcher, of Anna, Illinois, after passing a restless night from indisposition, got up and took a dose of the above-named medicine, about six o'clock, to produce rest. She had been in the habit of using it, but unfortunately took three dessert spoonfuls. Her husband, on going to her from breakfast, saw that something was wrong, and summoned aid, when she was found unconscious, and as nothing could be learned as to what she had taken, it was supposed to be some kind of an opiate. She was completely narcotized, although the pupil of the eye wanted that peculiar expression usually found when opium has been taken. Drs. Parks and Garlington were called in, and subsequently Dr. Schuchardt, of Jonesboro'. Antidotes, stimulants, and other agents, calculated to destroy the force of the poison, were used, resulting in her recovery, after more than eighteen hours of insensibility.—*Medical and Surgical Reporter.*

